# Associations between plasma metal elements and risk of cognitive impairment among Chinese older adults

**DOI:** 10.3389/fnagi.2024.1353286

**Published:** 2024-02-07

**Authors:** Xin Wang, Binbin Wang, Fuwen Yang, Kaijian Shang, Shaowei Chen, Yue Zhang

**Affiliations:** ^1^Department of Neurology, Tongji Shanxi Hospital, Shanxi Bethune Hospital, Shanxi Academy of Medical Sciences, Third Hospital of Shanxi Medical University, Taiyuan, China; ^2^School of Life Science, Shanxi Normal University, Taiyuan, China; ^3^Department of Emergency Medicine, Second Hospital of Shanxi Medical University, Taiyuan, China; ^4^Department of Hematology, First Hospital of Shanxi Medical University, Taiyuan, China; ^5^School of Public Health, Department of Epidemiology, Shanxi Medical University, Key Laboratory of Coal Environmental Pathogenicity and Prevention, Ministry Education, Taiyuan, China

**Keywords:** metal elements, risk, cognitive impairment, older adults, mini-mental state examination, CLHLS

## Abstract

**Background:**

The relationship between plasma metal elements and cognitive function is unclear, especially in extremely older individuals. This present study aimed to explore the association between plasma metal concentrations and the risk of cognitive impairment (CI) in Chinese extremely older adults.

**Methods:**

Individuals aged ≥90 years with plasm metal concentration data from the fifth wave of the 2008 Chinese Longitudinal Healthy Longevity Survey were included. Plasma selenium (Se), manganese (Mn), magnesium (Mg), calcium (Ca), iron (Fe), copper (Cu), and zinc (Zn) concentrations were measured using inductively coupled plasma optical emission spectroscopy. Cognitive function was assessed by the Chinese version of the mini-mental state examination.

**Results:**

The study enrolled 408 participants. Participants with CI had significantly lower plasma Se, Mn, and Fe levels and higher Ca levels than those with normal cognitive function (*p* < 0.05). Plasma Se, Mn, Ca, and Fe concentrations were significantly associated with CI risk in both single- and multiple-element logistic regression models. Additionally, the multiple-element model results showed that the adjusted odds ratios for CI were 0.042 (95% confidence interval 0.016–0.109), 0.106 (0.044–0.255), 7.629 (3.211–18.124) and 0.092 (0.036–0.233) for the highest quartiles compared to the lowest quartiles of Se, Mn, Ca, and Fe, respectively. Moreover, subgroup analyses by age, sex, and body mass index suggested a consistent significant correlation (*p* < 0.05).

**Conclusion:**

Therefore, decreased plasma Se, Mn, and Fe and increased plasma Ca levels were associated with CI risk in Chinese older adults. These findings are of great significance for the development of programs to delay cognitive decline in the elderly.

## 1 Introduction

Alzheimer’s disease (AD) is clinically characterized by progressive memory impairment and widespread cognitive function deterioration. The primary neuropathological hallmark of AD includes amyloid-β (Aβ) and hyperphosphorylated tau. In 2021, approximately 55 million people worldwide suffered from dementia, and this number is expected to triple by 2050 (Gauthier et al., 2021). In China, an estimated 15.07 million people aged 60 years and older live with dementia (Jia et al., 2020), and the national annual cost for the treatment of dementia is $167.74 billion (Jia et al., 2018). AD is a major public health problem that is becoming increasingly important worldwide.

AD is a multifactorial disorder involving genetics, environmental, and aging factors in its pathogenesis and progress. An increasing number of studies have implicated metal dysregulation in AD pathogenesis. Metals are ubiquitous in nature and can enter the human body via the gastrointestinal tract and air–blood tissue barrier in the lungs. Metals are necessary elements for the normal operation of cell homeostasis and life activities. As an integral component of many metalloenzymes, metals are involved in intra- and inter-neuronal signaling by modulating the functions of numerous enzymes (Das et al., 2021). Previous studies have shown that metals are associated with Aβ deposition and tau pathology (Wang et al., 2020; Chen et al., 2023). Furthermore, metals can cause mitochondrial dysfunction, subsequently decreased adenosine triphosphate and increased reactive oxygen species formation, and ultimately lead to neuronal cell death and neurological diseases (Cheng H et al., 2021). An imbalance in metals can also contribute to changes in blood–brain barrier (BBB) permeability (Zhu et al., 2018), neuroinflammation (Chen et al., 2023), endoplasmic reticulum stress (Wang et al., 2020), autophagic dysfunctions (Wang et al., 2020), oxidative stress (Greenough et al., 2013), and other effects common to AD.

Several previous studies have reported an association between metal element levels and AD, although the results have been inconsistent. He et al. performed a Mendelian randomization study on a large-scale genome-wide association study dataset assessing the relationship between serum calcium (Ca) and AD, which revealed that increased serum Ca was correlated with declined AD risk via inverse-variance weighing (He et al., 2020). In addition, some studies have shown that, compared with healthy controls, participants with AD have significantly reduced serum magnesium (Mg) (Du et al., 2022), manganese (Mn) (Du et al., 2017), iron (Fe) (Gong et al., 2021), and blood selenium (Se) (Cardoso et al., 2021) levels. Two recent studies in older adults without dementia reported that decreased serum zinc (Zn) (Kim et al., 2021) and copper (Cu) (Choe et al., 2022) levels can promote brain amyloid deposition and cognitive decline. These results suggest that metal deficiency may be a contributing factor in AD. However, other studies have found no association between plasma Mg (Thomassen et al., 2021) or serum Cu (Al-Khateeb et al., 2014) levels and AD. Some other studies have demonstrated that high serum Cu, Ca, and Fe levels increase AD risk. Ma et al. conducted a 10-year follow-up study of 1,224 elderly adults without dementia from the Alzheimer’s Disease Neuroimaging Initiative cohort and found that higher baseline plasma Ca levels were associated with a higher risk of developing AD than those with lower baseline plasma Ca levels (Ma et al., 2020). Squitti et al. performed a meta-analysis of 56 studies, and showed that participants with AD have higher serum Cu levels, which was associated with a three-four folds increase in the risk of AD (Squitti et al., 2021). Sternberg et al. analyzed the related Fe parameters of serum samples obtained from the Oregon Brain Tissue Bank and demonstrated that, compared to healthy controls, patients with AD had higher hepcidin and Fe-related protein, which were significantly associated with cognitive function (Sternberg et al., 2017). However, few studies have explored the relationship between multiple metal elements and CI risk, especially in the extremely older population.

As the main clinical manifestation of AD, cognitive impairment (CI) presents a significant challenge to China’s healthcare system because the nation has the world’s largest population of older adults with CI (Jia et al., 2020). China is a rapidly aging country, and exploration regarding factors affecting cognitive function among extremely elderly individuals is helpful for policymaking and the development of public health measures for healthy aging. Thus, the present study investigated the relationship between multiple plasma metal levels and CI risk in the extremely older population based on the data from the fifth wave of the 2008 Chinese Longitudinal Healthy Longevity Survey (CLHLS).

## 2 Materials and methods

### 2.1 Sample

The present study utilized data from the fifth wave of the 2008 CLHLS, an ongoing prospective cohort survey of 23 Chinese provinces for investigating the health of community-dwelling Chinese older people. The CLHLS was initiated in 1998 with follow-up intervals every 2–3 years. Surveys were performed via face-to-face interviews. A structured questionnaire that included sociodemographic characteristics, lifestyle, medical history, and physical examinations was administered by well-trained interviewers. Further details regarding the survey design and sample screening procedures have been described elsewhere (Gu et al., 2020). Survey data are freely accessible on the following site: https://opendata.pku.edu.cn/dataverse/CHADS.

**Figure 1:**
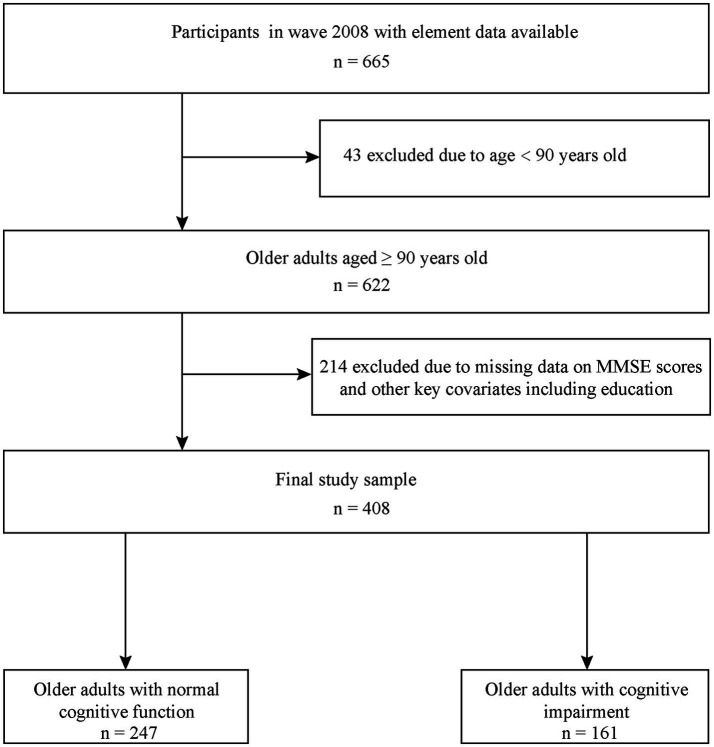
Detailed flowchart of participant selection. MMSE, mini-mental state examination.

This present study used a cross-sectional design among CLHLS participants ≥90 years of age in 2008. Plasma metal concentrations were first collected in the 2008 wave of the CLHLS. After excluding 43 individuals who were younger than 90 years and 214 individuals without mini-mental state examination (MMSE) data and other key covariates, 408 individuals were included. Figure 1 shows a detailed flowchart detailing the process of selection.

The CLHLS followed the Declaration of Helsinki and was approved by the Ethics Committee of Peking University (IRB00001052-13074). All participants signed a written informed consent.

### 2.2 Cognitive function assessment

The MMSE is widely used to evaluate cognitive function, with higher MMSE scores indicating better cognitive function. In the present study, cognitive function was evaluated using the Chinese version of the MMSE, which was translated from the English version, with small modifications based on the social differences of the Chinese population. The MMSE covers seven cognitive domains: orientation, attention, calculation, registration and recall, language capability, spatial ability, and executive function. Respondents answered all questions without a proxy. Considering that approximately 85% of our study participants had no formal education, participants with MMSE scores <18 were defined as having CI, as described in a previous study (Jin et al., 2021).

### 2.3 Plasma metal measurement

Briefly, plasma was isolated from whole blood by centrifugation at 3000 ×*g* for 10 min at room temperature. Plasma samples were then mixed with HNO_3_ and HClO_4_ (4:1 by volume) and digested overnight using an electric hot plate. After digestion, the residue was dissolved in 1% HNO_3_. The plasma metal concentrations were determined using previously established protocols by inductively coupled plasma optical emission spectroscopy (ICP-OES-7000, Shimadzu Co., Kyoto, Japan) (Shen et al., 2020).

### 2.4 Statistical analyses

Comparisons of variables were conducted using Student’s t-test or chi-squared tests when the data were normally distributed. Otherwise, non-parametric tests were used. We used logistic regression to evaluate the relationship between plasma metal levels and CI risk. Taking the correlation between the first quartile of plasma metal concentrations and the CI risk as a reference group, we evaluated the correlations between the second through the fourth quartiles and CI risk, which were expressed using odds ratios with 95% confidence intervals. We conducted a sensitivity analysis to check the robustness of our findings, by adjusting for lifestyle and medical history. For these elements significantly associated with CI risk in the multiple-element model, we further performed a logistic spline regression analysis to test dose–response relationship with CI risk. Subgroup analyses stratified by age, sex, and body mass index (BMI) were performed to evaluate the relationship between plasma metal elements and CI risk. In addition, a multiple-element model was used to evaluate the joint associations between elements significantly associated with CI risk. Statistical analyses were performed using SPSS 26.0 (IBM, Armonk, NY, United States) and R 3.6.1 software (R Foundation, Vienna, Austria), and a *p*-value less than 0.05 was considered statistically significant.

## 3 Results

### 3.1 Study participants

A total of 408 participants aged ≥90 years, including 161 with CI and 247 without, were included in the final analyses. There were no significant differences in age, sex, ethnicity, marital status, or years of schooling between participants with and without CI. In addition, no significant differences were found between the two groups regarding smoking and drinking history, regular physical activity, BMI, or medical history including hypertension, diabetes, stroke, cardiovascular disease, respiratory diseases, and cancer. Participants in the CI group had significantly lower plasma Se, Mn, and Fe concentrations and higher Ca levels than those in the without CI group. Table 1 lists the detailed demographic characteristics of all study participants.

**Table 1:** The detailed demographic and clinical characteristics of all participants.

Characteristics	Total (*n* = 408)	NC group (*n* = 247)	CI group (*n* = 161)	*p*-value
Age (years), mean (SD)	97.10 (4.92)	97.32 (4.85)	96.75 (5.03)	0.246
Men (vs. women), *n* (%)	86 (21.08)	52 (21.05)	34 (21.12)	0.987
Ethnicity (Han vs. others), *n* (%)	344 (84.31)	208 (84.21)	136 (84.47)	0.943
Marital status, *n* (%)				0.342
Married	26 (6.37)	19 (7.69)	7 (4.35)	
Widowed	374 (91.67)	224 (90.69)	150 (93.17)	
Other	8 (1.96)	4 (1.62)	4 (2.48)	
Years of schooling, mean (SD)	0.47 (0.07)	0.45 (0.09)	0.50 (0.12)	0.756
Smoking history (yes vs. no), *n* (%)	89 (21.81)	57 (23.08)	32 (19.88)	0.444
Drinking history (yes vs. no), *n* (%)	86 (21.08)	54 (21.86)	32 (19.88)	0.631
Regular physical activity (yes vs. no), *n* (%)	94 (23.04)	65 (26.32)	29 (18.01)	0.052
BMI (kg/m^2^)	19.21 (2.84)	19.33 (2.87)	19.03 (2.79)	0.302
Hypertension (yes vs. no), *n* (%)	52 (12.75)	33 (13.36)	19 (11.80)	0.644
Diabetes (yes vs. no), *n* (%)	11 (2.70)	5 (2.02)	6 (3.73)	0.299
Stroke (yes vs. no), *n* (%)	7 (1.72)	4 (1.62)	3 (1.86)	0.853
Cardiovascular disease (yes vs. no), *n* (%)	17 (4.17)	11 (4.45)	6 (3.73)	0.720
Respiratory diseases (yes vs. no), *n* (%)	28 (6.86)	15 (6.07)	13 (8.07)	0.434
Cancer (yes vs. no), *n* (%)	9 (2.21)	6 (2.43)	3 (1.86)	0.704
Selenium [M, (P_25_, P_75_) μg/mL]	113.1 (81.85, 154.1)	122.1 (93.61, 162.8)	98.33 (73.24, 140.9)	**0.002**
Manganese [M, (P_25_, P_75_) μg/mL]	0.027 (0.012, 0.062)	0.034 (0.016, 0.073)	0.021 (0.009, 0.041)	**<0.001**
Magnesium [M, (P_25_, P_75_) μg/mL]	25.04 (20.30, 29.15)	24.20 (19.53, 29.02)	25.70 (21.32, 29.79)	0.199
Calcium [M, (P_25_, P_75_) μg/mL]	129.9 (98.44, 157.2)	122.0 (91.78, 151.6)	140.3 (107.9, 165.9)	**0.006**
Iron [M, (P_25_, P_75_) μg/mL]	3.829 (1.842, 7.645)	4.498 (2.122, 8.574)	3.406 (1.571, 6.341)	**0.001**
Copper [M, (P_25_, P_75_) μg/mL]	1.288 (1.047, 1.596)	1.296 (1.055, 1.601)	1.264 (1.015, 1.582)	0.488
Zinc [M, (P_25_, P_75_) μg/mL]	2.034 (1.084, 3.197)	2.005 (0.971, 3.470)	2.072 (1.112, 3.034)	0.785

Significant results appear in bold. CI, cognitive impairment; BMI, body mass index; SD, standard deviation; M, median; P25, 25% quartile; P75, 75% quartile.

### 3.2 Plasma elements and CI risk

**Figure 2:**
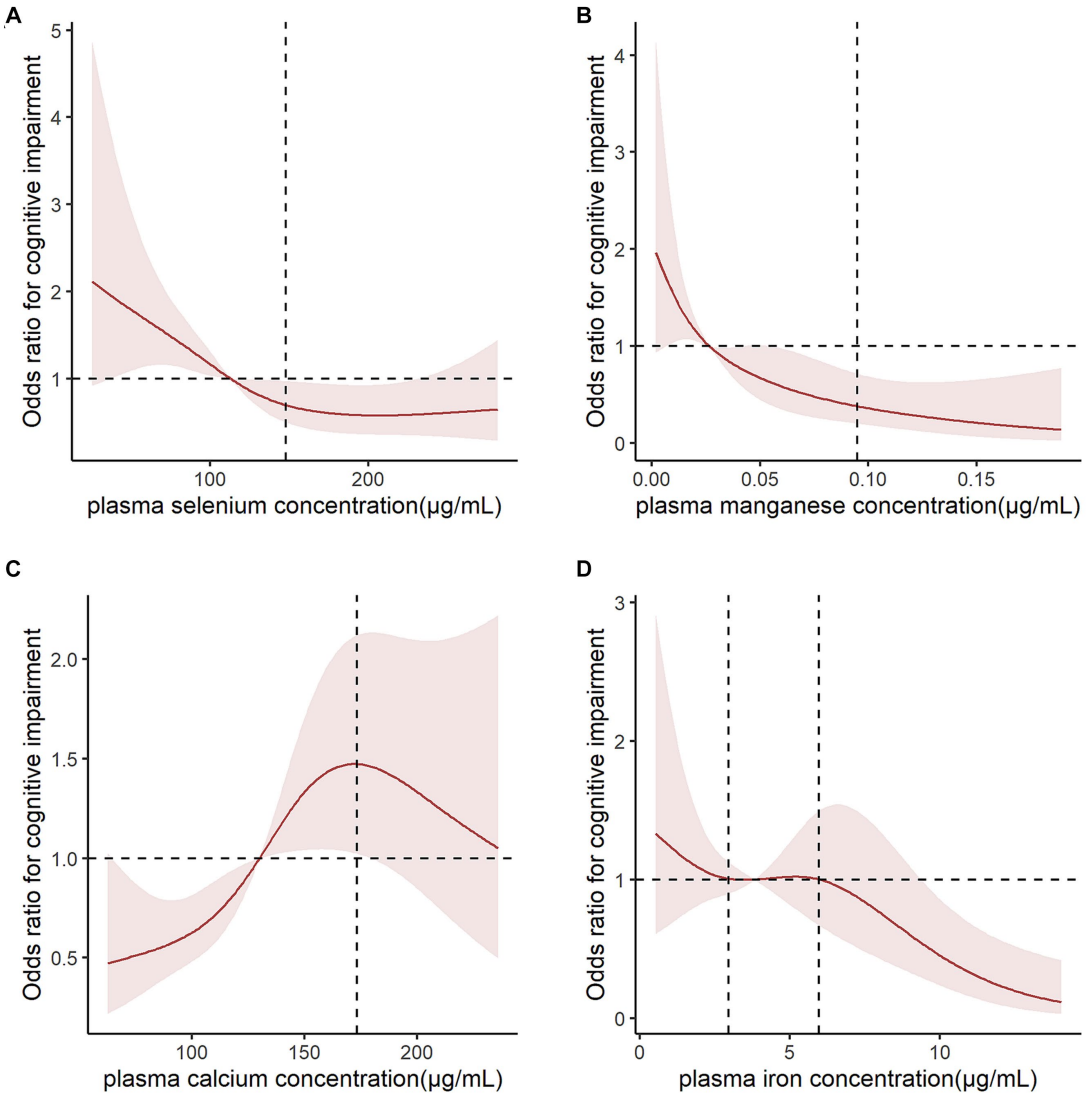
Logistic spline regression analysis for testing the dose–response relationship between metal levels and cognitive impairment risk. Odds ratios (ORs) for cognitive impairment (CI) declined dramatically when **(A)** plasma selenium concentrations were <147.85 μg/mL and **(B)** plasma magnesium concentrations were <0.095 μg/mL. **(C)** Plasm calcium concentration had an inverted U-shaped association with CI risk, with 173.16 μg/mL as an inflection point. **(D)** When plasma iron concentrations were between 2.961–5.969 μg/mL, the OR did not change significantly. The results were adjusted for age, sex, ethnicity, marital status, education, and body mass index.

The results of single-element (Supplementary Table S1) and multiple-element (Table 2) models suggested that plasma Se, Mn, Ca, and Fe levels were significantly correlated with CI risk after adjusting for confounding factors (*p* < 0.05). The sensitivity analysis suggested consistent associations between these metal elements and CI risk and confirmed the robustness of our findings (Table 3). We further performed a logistic spline regression analysis to test the dose–response relationship between metal elements levels and CI risk. The correlations between Se, Mn, Ca, and Fe levels and CI risk were non-linear (*p* > 0.05). The odds ratio (OR) for CI declined dramatically with increasing Se concentrations when the plasma Se concentrations were < 147.85 μg/mL. When plasma Se concentrations were ≥ 147.85 μg/mL, no evident OR change was observed. CI risk had a downward trend with increasing Mn concentration. The OR for CI declined dramatically when the plasma Mn concentration was <0.095 μg/mL. Interestingly, the plasma Ca concentration had an inverted U-shaped association with CI risk, with 173.16 μg/mL as an inflection point. When plasma Fe concentrations were between 2.961–5.969 μg/mL, the OR did not change significantly (Figure 2).

**Table 2:** Odds ratios (95% confidence interval) for cognitive impairment based on plasma elements concentration interquartile range in the multiple-element models.

Plasma elements	Odds ratios (95% confidence interval)				*p*-value
	Q1	Q2	Q3	Q4	
Selenium (μg/mL)	2.719–81.793	82.001–112.834	113.3–153.887	154.186–375.705	
Model 1	1	0.315 (0.174, 0.569)	0.334 (0.183, 0.612)	0.224 (0.117, 0.427)	**<0.001**
Model 2	1	0.140 (0.066, 0.300)	0.146 (0.067, 0.316)	0.042 (0.016, 0.109)	**<0.001**
Manganese (μg/mL)	0.001–0.012	0.012–0.027	0.027–0.061	0.062–2.714	
Model 1	1	0.532 (0.300, 0.943)	0.540 (0.303, 0.961)	0.180 (0.093, 0.346)	**<0.001**
Model 2	1	0.459 (0.229, 0.919)	0.451 (0.225, 0.904)	0.106 (0.044, 0.255)	**<0.001**
Calcium (μg/mL)	14.701–98.358	98.692–129.865	129.989–157.142	157.216–344.156	
Model 1	1	1.873 (1.019, 3.443)	2.322 (1.270, 4.246)	3.185 (1.747, 5.804)	**0.002**
Model 2	1	3.886 (1.750, 8.629)	3.272 (1.445, 7.408)	7.629 (3.211, 18.124)	**<0.001**
Iron (μg/mL)	0.337–1.838	1.857–3.819	3.839–7.631	7.650–19.993	
Model 1	1	0.680 (0.387, 1.197)	0.846 (0.478, 1.496)	0.243 (0.126, 0.469)	**<0.001**
Model 2	1	0.350 (0.167, 0.730)	0.430 (0.201, 0.922)	0.092 (0.036, 0.233)	**<0.001**

Significant results appear in bold. Plasma elements which were significant in the single-element were included in this multiple-element models. Model 1: Plasma elements were included in the single-element model and adjusted for age, sex, ethnicity, marital status, education, and body mass index. Model 2: Plasma elements were included in multiple-elements model and adjusted for the same variables as Model 1.

**Table 3:** Sensitive analysis for the associations between plasma elements and the risk of cognitive impairment.

Plasma elements	Odds ratios (95% confidence interval)				*p*-value
	Q1	Q2	Q3	Q4	
Selenium (μg/mL)	2.719–81.793	82.001–112.834	113.3–153.887	154.186–375.705	
Model 1	1	0.140 (0.066, 0.300)	0.146 (0.067, 0.316)	0.042 (0.016, 0.109)	**<0.001**
Model 2	1	0.155 (0.074, 0.323)	0.165 (0.079, 0.345)	0.058 (0.024, 0.138)	**<0.001**
Model 3	1	0.135 (0.063, 0.291)	0.161 (0.076, 0.343)	0.056 (0.023, 0.135)	**<0.001**
Model 4	1	0.126 (0.058, 0.275)	0.157 (0.074, 0.336)	0.051 (0.021, 0.126)	**<0.001**
Manganese (μg/mL)	0.001–0.012	0.012–0.027	0.027–0.061	0.062–2.714	
Model 1	1	0.459 (0.229, 0.919)	0.451 (0.225, 0.904)	0.106 (0.044, 0.255)	**<0.001**
Model 2	1	0.472 (0.242, 0.921)	0.474 (0.244, 0.919)	0.121 (0.054, 0.271)	**<0.001**
Model 3	1	0.402 (0.203, 0.798)	0.421 (0.213, 0.832)	0.092 (0.040, 0.213)	**<0.001**
Model 4	1	0.391 (0.195, 0.784)	0.415 (0.209, 0.824)	0.090 (0.038, 0.210)	**<0.001**
Calcium (μg/mL)	14.701–98.358	98.692–129.865	129.989–157.142	157.216–344.156	
Model 1	1	3.886 (1.750, 8.629)	3.272 (1.445, 7.408)	7.629 (3.211, 18.124)	**<0.001**
Model 2	1	3.601 (1.676, 7.738)	2.674 (1.280, 5.582)	6.186 (2.919, 13.109)	**<0.001**
Model 3	1	3.905 (1.762, 8.654)	2.932 (1.365, 6.299)	6.852 (3.024, 14.323)	**<0.001**
Model 4	1	3.903 (1.748, 8.714)	2.966 (1.371, 6.416)	6.977 (3.171, 15.354)	**<0.001**
Iron (μg/mL)	0.337–1.838	1.857–3.819	3.839–7.631	7.650–19.993	
Model 1	1	0.350 (0.167, 0.730)	0.430 (0.201, 0.922)	0.092 (0.036, 0.233)	**<0.001**
Model 2	1	0.335 (0.165, 0.684)	0.405 (0.197, 0.836)	0.095 (0.040, 0.225)	**<0.001**
Model 3	1	0.263 (0.125, 0.557)	0.335 (0.158, 0.708)	0.073 (0.030, 0.178)	**<0.001**
Model 4	1	0.249 (0.115, 0.536)	0.314 (0.146, 0.677)	0.067 (0.027, 0.168)	**<0.001**

Significant results appear in bold. Model 1: Plasma elements were included in the multiple-elements models and adjusted for age, sex, ethnicity, marital status, education, and body mass index. Model 2: Adjusted as in Model 1 and additionally adjusted for smoking and drinking status. Model 3: Adjusted as in Model 2 and additionally adjusted for regular physical activity. Model 4: Adjusted as in Model 3 and additionally adjusted for medical history including hypertension, diabetes, stroke, cardiovascular disease, respiratory diseases, and cancer.

### 3.3 Subgroup and element interaction analyses

Supplementary Figure S1 shows the link between plasma metal elements and CI risk in three subgroups stratified by age, sex, and BMI. The results still demonstrated that plasma Se, Mn, Ca, and Fe levels were correlated with CI risk (*p* < 0.05). This correlation was stronger among males than females. In addition, we found that the three metal elements of Se, Mn, and Fe have synergistic effects with each other. However, an increase in the Ca concentration counteracted the protective effects of these three elements on cognitive function (Supplementary Table S2).

## 4 Discussion

In this cross-sectional study of Chinese older adults aged ≥90 years, we explored the associations between plasma metals and CI risk. The results indicated that plasma Mn, Fe, and Zn levels were negatively correlated, and Ca was positively correlated with CI risk. In addition, we found synergistic effects for CI risk between Se, Mn, and Fe. Se, an essential trace element, is an important cofactor for the antioxidant enzyme glutathione peroxidase, which exhibits neuroprotection by reducing oxidative stress in the brain. Our study suggests that the plasma Se concentration was significantly lower in older adults aged ≥90 years with CI. This is consistent with a previous study (Zhou et al., 2023). A recent study demonstrated that higher serum Se levels are linked to decreased serum Aβ42 and Aβ40 (Luo et al., 2023), proposing a potential neuroprotective action of Se. However, studies have also been conducted from different perspectives. A case–control study by Gu et al. (2021) suggested that increased Se concentration was correlated with an increased CI risk. Vinceti et al. (2022) found that the Se concentration in the cerebrospinal fluid was inversely correlated with hippocampal volume, suggesting Se may have some role in the development of AD. Nevertheless, recent research indicates that increasing dietary Se intake has a positive effect on cognitive function (Pereira et al., 2022). The beneficial role of Se in cognition has been demonstrated in several animal studies. A study using an AD transgenic mouse model showed that dietary supplementation with Se-enriched yeast for 3 months significantly suppressed inflammatory processes, reversed synaptic dysfunction, inhibited tau phosphorylation, and ultimately improved cognitive function (Zhang et al., 2017). Liang et al. (2023) reported that, in a mouse model of neuroinflammation, Se supplementation inhibited neural cell death and improved cognitive function in the mouse model, by an increased expression of selenoproteins. In addition, some previous studies have shown that Se has a beneficial role in AD pathogenesis by reducing mitochondrial oxidative stress (Farbood et al., 2020), attenuating Aβ aggregation, and reducing tau hyperphosphorylation (Li et al., 2018).

Our study suggest that plasma Mn levels were significantly lower in older adults with CI, which is consistent with a previous study (Du et al., 2017). However, the contribution of Mn reduction to AD progression remains unclear. It is well known that Mn serves as a cofactor for many enzymes, including glutamine synthetase, phosphoenolpyruvate decarboxylase, and Mn superoxide dismutase (SOD). These metalloproteins are crucial in the processes of antioxidative stress, energy metabolism, and immune function; thus, we infer that the dysfunction of these enzymes may precipitate AD development (Du et al., 2017). Balendra and Singh (2021) showed that Mn-SOD decreases Bace1 expression while inhabiting the deposition of Aβ plaque and tau protein. SOD supplementation prevented cognitive decline by reducing lipid peroxidation and maintaining hippocampal neurogenesis in mice models. In addition, studies have reported the dysfunction of Mn transporters in AD mice and patients (Tian et al., 2018). We infer that the decreased plasma Mn in the CI group may have been the result of dysfunctional Mn transporters, which can maintain Mn homeostasis (Kumar et al., 2019).

Our data showed an inverted-U relationship between plasma Ca levels and CI risk in older adults aged ≥90 years. Ca is an indispensable secondary messenger that regulates many processes in cells including gene transcription, cell proliferation, migration, and apoptosis (Baracaldo-Santamaría et al., 2023). It is well-known that excessive Ca influx induces reactive oxygen species overproduction correlated with neuronal death (Sugiyama et al., 2017). Elevated extracellular Ca levels lead to Ca influx, increased intracellular Ca levels, and neuronal death via Ca-sensing receptors. In addition, Aβ and tau are involved in this process. Higher Ca ion concentrations are associated with increased Aβ accumulation and tau hyperphosphorylation (Guan et al., 2021). Ma et al. (2020) reported that plasma Ca was positively correlated with cerebrospinal fluid Aβ pathology. Meanwhile, preclinical evidence suggests that Aβ activates the cell membrane receptors, forms Ca- permeable ion channels, and increases intracellular Ca levels, finally leading to neuron death (Tong et al., 2018). Additionally, a longitudinal population-based study showed that Ca supplementation increased AD risk (Kern et al., 2016). Our study shows ORs decreasing with increasing Ca concentrations when plasma Ca concentrations were ≥ 173.16 μg/mL. The reason for this correlation between higher plasma Ca (≥173.16 μg/mL) and decreased cognitive decline remains unclear. We presume that the survivor effect may explain this. Previous research has shown that high plasma Ca can cause various complications including hyperlipidemia, coronary heart disease, myocardial infarction, ischemic stroke, and cancer (Larsson et al., 2017; Cheng J et al., 2021; Kobylecki et al., 2021; Yun et al., 2022), ultimately leading to an increase in mortality. Therefore, we infer that many people may die from various comorbidities caused by higher plasma Ca levels among older adults with higher plasma Ca levels and thus there are fewer older adults surviving to experience CI. This may be why CI risk had a downward trend with increasing plasma Ca concentration.

This present study showed that compared to participants in the normal cognition group, the CI group participants had lower plasma Fe levels, which is consistent with previous studies (Gong et al., 2021). In addition, in a 2-year follow-up study, Jakubowski et al. (2021) found that serum Fe levels significantly decreased in mild CI as the disease progressed. Thus, we inferred that Fe dysfunction may be relevant to AD pathogenesis and progress. Fe is critical for cell growth, differentiation, and the maintenance of cell identity (Babić Leko et al., 2023). In addition, Kim and Connor (2020) suggested that neurotransmitter and myelin synthesis and synapse formation require adequate and timely Fe supplementation. Crespo et al. (2014) demonstrated that serum Fe transporter and storage proteins were significantly decreased in patients with AD. In addition, downregulated serum Fe levels in AD could be due to increased Fe-regulatory hormone (hepcidin) levels (Kweon et al., 2019). Several studies in animals have demonstrated the beneficial role of Fe supplementation in improving cognitive function. Shen et al. (2019) found that mice with Fe-supplemented drinking water had a dramatic decrease in the levels of Aβ_42_, phosphorylated tau, and neuronal apoptosis. However, one study reported that cortical Fe levels are increased in patients with AD and co-localized with Aβ plaques (Jakubowski et al., 2021). This may be due to peripheral Fe deficiency, leading to Fe transporters in the BBB taking up more Fe2+ and releasing it in the brain, ultimately resulting in Fe overload, neuroinflammation, and Aβ deposition (Dong et al., 2015).

There are several strengths to our present study. First, this study explored the association between plasma metal elements and CI risk in Chinese older adults for the first time. Second, the representative high-quality CLHLS database was used and adjusted for confounding factors in the regression models. Third, we performed further subgroup analyses to investigate the relationship between plasma elements and CI risk. However, several limitations should be considered when interpreting our results. The plasma metal element levels measured in our study were the reflection of recent exposure instead of long-term exposure. In a future study, we will measure the metal element levels from hair and/or nails, which indicate the long-term metal burden (Koseoglu et al., 2017), and analyze the association between them and CI risk. In addition, causal associations between plasma metals and CI risk could not be determined because this was a cross-sectional study. A longitudinal follow-up of participants is needed to explore the metal elements levels and the likelihood of incidence of CI.

## 5 Conclusion

In conclusion, decreased plasma Se, Mn, and Fe levels and increased plasma Ca levels were associated with CI risk in Chinese older adults aged ≥90 years. These findings may be valuable for providing dietary modification and improving cognitive function in Chinese older adults.

## Data availability statement

Publicly available datasets were analyzed in this study. This data can be found here: https://opendata.pku.edu.cn/dataverse/CHADS.

## Ethics statement

The studies involving humans were approved by the Ethics Committee of Peking University (IRB00001052-13074). The studies were conducted in accordance with the local legislation and institutional requirements. Written informed consent for participation in this study was provided by the participants’ legal guardians/next of kin.

## Author contributions

XW: Conceptualization, Data curation, Formal analysis, Funding acquisition, Methodology, Supervision, Writing – original draft, Writing – review & editing. BW: Data curation, Formal analysis, Funding acquisition, Validation, Writing – review & editing. FY: Data curation, Formal analysis, Validation, Writing – original draft. KS: Data curation, Formal analysis, Validation, Writing – original draft. SC: Data curation, Formal analysis, Validation, Writing – original draft. YZ: Methodology, Software, Writing – original draft.
