# Supplementary material for: Associations between plasma metal elements and risk of cognitive impairment among Chinese older adults
Source: Front Aging Neurosci. 2024 Feb 7;16:1353286. doi: 10.3389/fnagi.2024.1353286 (PMC10879289; doi:10.3389/fnagi.2024.1353286)
Supplement: Supplementary file 1 [file Table_1.DOCX]

**Table S1.** Odds ratios (95% confidence interval) for cognitive impairment based on plasma elements concentration interquartile range in the single element models

| Plasma elements | Odds ratios (95% confidence interval) | | | | | | |
| --- | --- | --- | --- | --- | --- | --- | --- |
|  | Selenium | Manganese | Magnesium | Calcium | Iron | Copper | Zinc |
| Q1 | 2.719-81.793 | 0.001-0.012 | 7.739-20.294 | 14.701-98.358 | 0.337-1.838 | 0.152-1.047 | 0.044-1.084 |
| Odds ratio (95%CI) | 1 | 1 | 1 | 1 | 1 | 1 | 1 |
| Q2 | 82.001-112.834 | 0.012-0.027 | 20.303-25.027 | 98.692-129.865 | 1.857-3.819 | 1.048-1.287 | 1.084-2.030 |
| Odds ratio (95%CI) | 0.315(0.174,0.569) | 0.532(0.300,0.943) | 1.675(0.913,3.076) | 1.873(1.019,3.443) | 0.680(0.387,1.197) | 0.876(0.495,1.550) | 1.272(0.714,2.266) |
| *P* | <0.001 | 0.031 | 0.096 | 0.043 | 0.182 | 0.650 | 0.415 |
| Q3 | 113.3-153.887 | 0.027-0.061 | 25.044-29.152 | 129.989-157.142 | 3.839-7.631 | 1.288-1.594 | 2.037-3.183 |
| Odds ratio (95%CI) | 0.334(0.183,0.612) | 0.540(0.303,0.961) | 2.020(1.108,3.681) | 2.322(1.270,4.246) | 0.846(0.478,1.496) | 0.767(0.432,1.363) | 1.681(0.941,3.003) |
| *P* | <0.001 | 0.036 | 0.022 | 0.006 | 0.565 | 0.366 | 0.079 |
| Q4 | 154.186-375.705 | 0.062-2.714 | 29.152-78.319 | 157.216-344.156 | 7.650-19.993 | 1.596-10.945 | 3.202-10.293 |
| Odds ratio (95%CI) | 0.224(0.117,0.427) | 0.180(0.093,0.346) | 1.748(0.969,3.153) | 3.185(1.747,5.804) | 0.243(0.126,0.469) | 0.790(0.440,1.417) | 0.978(0.540,1.770) |
| *P* | <0.001 | <0.001 | 0.064 | <0.001 | <0.001 | 0.429 | 0.940 |
| *P*-VALUE | **<0.001** | **<0.001** | 0.118 | **0.002** | **<0.001** | 0.803 | 0.212 |

*NOTE.* Plasma elements were included in the single-element model and adjusted for age, sex, ethnicity, marital status, education, and body mass index.
