# Supplementary material for: Associations between plasma metal elements and risk of cognitive impairment among Chinese older adults
Source: Front Aging Neurosci. 2024 Feb 7;16:1353286. doi: 10.3389/fnagi.2024.1353286 (PMC10879289; doi:10.3389/fnagi.2024.1353286)
Supplement: Supplementary file 2 [file Table_2.DOCX]

**Table S2.** Odds ratios (95% confidence interval) for CI according to the combined categories of plasma element levels

| Plasma elements | Odds ratio (95% confidence interval) | *P*-value |
| --- | --- | --- |
| Selenium- Manganese |  |  |
| Low Se + Low Mn | 1 | **<0.001** |
| High Se + Low Mn | 0.303(0.163,0.563) | **<0.001** |
| Low Se + High Mn | 0.270(0.144,0.504) | **<0.001** |
| High Se + High Mn | 0.192(0.097,0.377) | **<0.001** |
| Selenium- Calcium |  |  |
| Low Se + Low Ca | 1 | **<0.001** |
| High Se + Low Ca | 0.685(0.369,1.269) | 0.229 |
| Low Se + High Ca | 2.417(1.365,4.279) | **0.002** |
| High Se + High Ca | 1.049(0.572,1.923) | 0.877 |
| Selenium-Iron |  |  |
| Low Se + Low Fe | 1 | **<0.001** |
| High Se + Low Fe | 0.259(0.138,0.488) | **<0.001** |
| Low Se + High Fe | 0.299(0.159,0.563) | **<0.001** |
| High Se + High Fe | 0.253(0.128,0.501) | **<0.001** |
| Manganese- Calcium |  |  |
| Low Mn + Low Ca | 1 | **<0.001** |
| High Mn + Low Ca | 0.347(0.186,0.646) | **0.001** |
| Low Mn + High Ca | 1.344(0.758,2.384) | 0.311 |
| High Mn + High Ca | 0.907(0.491,1.675) | 0.755 |
| Manganese-Iron |  |  |
| Low Mn + Low Fe | 1 | **0.001** |
| High Mn + Low Fe | 0.478(0.261,0.876) | **0.017** |
| Low Mn + High Fe | 0.650(0.356,1.186) | 0.160 |
| High Mn + High Fe | 0.311(0.171,0.566) | **<0.001** |
| Calcium- Iron |  |  |
| Low Ca + Low Fe | 1 | **0.001** |
| High Ca + Low Fe | 1.837(1.038,3.251) | **0.037** |
| Low Ca + High Fe | 0.508(0.269,0.957) | **0.036** |
| High Ca + High Fe | 1.177(0.663,2.091) | 0.577 |

*NOTE.* Significant results appear in bold.

Plasma elements which were significant in multiple-element models were included in the combined effect analysis. And the multiple-element model adjusted for age, sex, ethnicity, marital status, education, and BMI. The plasma elements concentration is limited by the median of the elements.

*Abbreviations: CI,* Cognitive impairment; *Se,* Selenium; *Mn,* Manganese; *Ca,* Calcium; *Fe,* Iron.
