# Supplementary material for: Associations between plasma metal elements and risk of cognitive impairment among Chinese older adults
Source: Front Aging Neurosci. 2024 Feb 7;16:1353286. doi: 10.3389/fnagi.2024.1353286 (PMC10879289; doi:10.3389/fnagi.2024.1353286)
Supplement: Supplementary file 3 [file Table_3.DOCX]

| **A** General demographic characteristics | |  | Code |
| --- | --- | --- | --- |
| A1 sex： | 1. male 2. female |  | □ |
| A2 ethnic group： | 1.Han 2. Hui 3. Zhuang 4. Yao  5.Korea 6. Man 7.Mongolia 8.others |  | □ |
| A3 age declared by the interviewee？  A3-2 date of birth： | ________years  ________ |  | □□□        |

| * C1 Orientation | | | | Code |
| --- | --- | --- | --- | --- |
| C1-1 what is the animal year of this year? | 1. yes 0. no | |  | □ |
| C1-2 What month is it today? | 1. yes 0. no | |  | □ |
| C1-3 what is the date today? | 1. yes 0. no | |  | □ |
| C1-4 What day is it today? | 1. yes 0. no | |  | □ |
| C1-5 what is the season right now? | 1. yes 0. No | |  |  |
| C1-6 what is the name of this province? | 1. yes 0. No | |  |  |
| C1-7 what is the name of this city? | 1. yes 0. No | |  |  |
| C1-8 what is the name of this county or district? | 1. yes 0. no | |  |  |
| C1-9 What floor are we on now? |  | |  |  |
| C1-10 where exactly are we now? | _____ | |  | □□ |
| * C2 Name | | |  | Code |
| C2-1  repeat the name of "table" at the first time  repeat the name of "apple" at the first time  repeat the name of "clothes" at the first time | 1. yes 0. no  1. yes 0. no  1. yes 0. no | |  | □  □  □ |
| * C3 Attention and calculation | | |  | Code |
| C3-1 20-3  20-3-3  20-3-3-3  20-3-3-3-3  20-3-3-3-3-3? | 1. yes 0. no  1. yes 0. no  1. yes 0. no  1. yes 0. no  1. yes 0. no | |  | □  □  □  □  □ |
| C3-2 draw the figure following the sample | 1. yes 0. no | |  | □ |
| * **C4** Recall | |  | | Code |
| C4-1 repeat the name of "table" a while later  repeat the name of "apple" a while later  repeat the name of "clothes" a while later | 1. yes 0. no  1. yes 0. no  1. yes 0. no |  | | □  □  □ |
| * C5 Language and executive capability | | | | Code |
| C5-1 naming "pen"  naming "watch" | 1. yes 0. no  1. yes 0. no |  | | □  □ |
| C5-2 repeat a sentence  “forty-four stone lions”  C5-2-1 act as the meaning of a sentence  **“Close your eyes”**  C5-2-2 write a complete sentence | 1. yes 0. no  1. yes 0. no  1. yes 0. no |  | | □  □  □ |
| C5-3 taking paper using right hand  folding paper  put paper on the floor | 1. yes 0. no  1. yes 0. no  1. yes 0. no |  | | □  □  □ |

| D Life style |  |  | Code |
| --- | --- | --- | --- |
| D7-1 smoke or not at present? | 1. yes 0. no | ○ | □ |
| D7-2 smoked or not in the past? | 1. yes 0. no | ○ | □ |
| D8-1 drink or not at present? | 1. yes 0. no | ○ | □ |
| D8-2 drank or not in the past? | 1. yes 0. no | ○ | □ |
| D9-1 exercise or not at present? | 1. yes 0. no | ○ | □ |
| D9-2 exercised or not in the past? | 1. yes 0. no | ○ | □ |

| F Personal background | |  | Code |
| --- | --- | --- | --- |
| F1 Years of schooling? | _______years | ○ | □□ |
| F4-1 marital status： | 1. Married  2. Divorced  3. Widowed  4. single | ○ | □ |
