# Supplementary figures and images for: Associations between plasma metal elements and risk of cognitive impairment among Chinese older adults
Source: Front Aging Neurosci. 2024 Feb 7;16:1353286. doi: 10.3389/fnagi.2024.1353286 (PMC10879289; doi:10.3389/fnagi.2024.1353286)

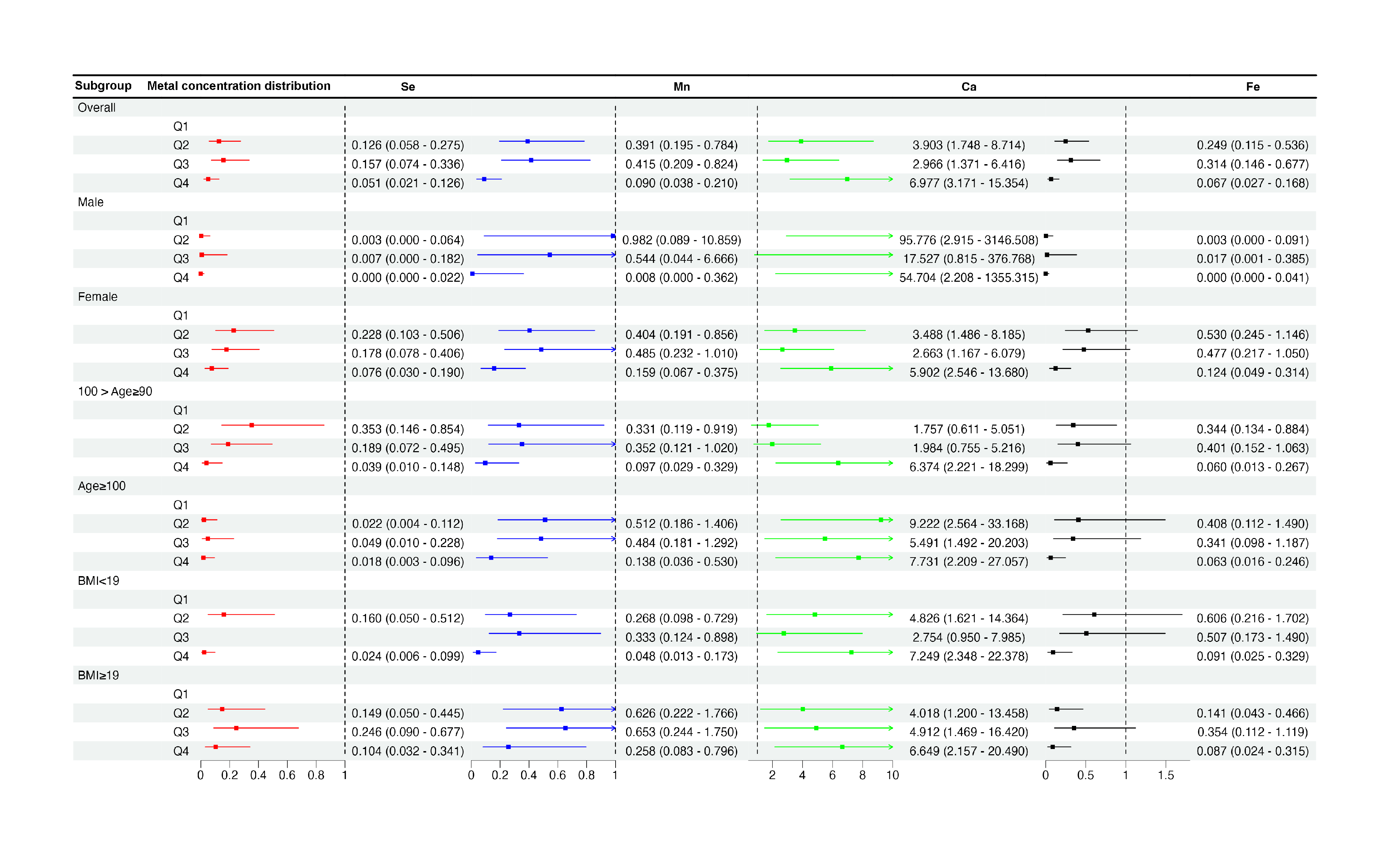

Supplement: Supplementary file 4 [file Image_1.TIF]
